# A Cross-Linker-Based Poly(Ionic Liquid) for Sensitive Electrochemical Detection of 4-Nonylphenol

**DOI:** 10.3390/nano9040513

**Published:** 2019-04-02

**Authors:** Jian Hu, Hao Dai, Yanbo Zeng, Yiwen Yang, Hailong Wang, Xudong Zhu, Lei Li, Guobao Zhou, Ruoyu Chen, Longhua Guo

**Affiliations:** 1 School of Petrochemical Engineering, Changzhou University, Changzhou 213016, China; 15189761689@163.com (J.H.); 13257926215@163.com (H.D.); 2 College of Biological, Chemical Sciences and Engineering, Jiaxing University, Jiaxing 314001, China; yangyiwen@mail.zjxu.edu.cn (Y.Y.); wanghailong@mail.zjxu.edu.cn (H.W.); zhuxudong9zxd@163.com (X.Z.); gbzhou@mail.zjxu.edu.cn (G.Z.); 3 MOE Key Laboratory for Analytical Science of Food Safety and Biology, Fujian Provincial Key Laboratory of Analysis and Detection Technology for Food Safety, Institute of Nanomedicine and Nanobiosensing, College of Chemistry, Fuzhou University, Fuzhou 350116, China; guolh@fzu.edu.cn

**Keywords:** poly(ionic liquid), cross-linker, electrochemical detection, 4-nonylphenol

## Abstract

In this study, we report a cross-linker-based poly(ionic liquid) (PIL) for the sensitive detection of 4-nonylphenol (4-NP). PIL was poly(1,4-butanediyl-3,3′-bis-l-vinylimidazolium dibromide) (poly([V_2_C_4_(mim)_2_]Br_2_)). Poly([V_2_C_4_(mim)_2_]Br_2_) was prepared via one-step free-radical polymerization. The poly([V_2_C_4_(mim)_2_]Br_2_) was characterized by infrared spectroscopy, Raman spectroscopy, thermal gravimetric analyzer and scanning electron microscope. The poly([V_2_C_4_(mim)_2_]Br_2_) was then drop-cast onto a glassy carbon electrode (GCE) to obtain poly([V_2_C_4_(mim)_2_]Br_2_)/GCE. In comparison with a bare GCE, poly([V_2_C_4_(mim)_2_]Br_2_)/GCE exhibited higher peak current responses for [Fe(CN)_6_]^3−/4−^, lower charge transfer resistance, and larger effective surface area. While comparing the peak current responses, we found the peak current response for 4-NP using poly([V_2_C_4_(mim)_2_]Br_2_)/GCE to be 3.6 times higher than a traditional cross-linker ethylene glycol dimethacrylate (EGDMA) based poly(EGDMA) modified GCE. The peak current of poly([V_2_C_4_(mim)_2_]Br_2_) sensor was linear to 4-NP concentration from 0.05 to 5 μM. The detection limit of 4-NP was obtained as 0.01 μM (S/N = 3). The new PIL based electrochemical sensor also exhibited excellent selectivity, stability, and reusability. Furthermore, the poly([V_2_C_4_(mim)_2_]Br_2_)/GCE demonstrated good 4-NP detection in environmental water samples.

## 1. Introduction

Nonylphenol ethoxylates are widely used for manufacturing various commercial products such as detergents, lubricating oil, paints, pesticides, and emulsifier, etc [1]. Nonylphenol ethoxylates degrade to produce 4-nonylphenol (4-NP) in an aquatic environment [2]. Since 4-NP can be produced industrially, naturally and through biodegradation of alkylphenol ethoxylates, the high prevalence of 4-NP in environment has been a grave concern due to its ability to mimic estrogen activity [2]. It is a well-known endocrine disruptor and xenoestrogen, causing serious harm to the reproductive health of human and wildlife [3]. Therefore, detection of 4-NP is of great significance. Various analytical methods including enzyme-linked immunosorbent assay [4], liquid chromatography [5], gas chromatography [6], fluorescence analysis [7] and electrochemical method [8] have been developed for the determination of 4-NP. Among these methods, the electrochemical method has received an increasing attention due to its many advantages such as time-saving, low cost, high sensitivity, and real-time detection. 

Poly(ionic liquids)s (PILs), a sub-class of polyelectrolytes, are polymeric materials in which the polymer backbone contains an ionic liquid (IL) species in each repeating unit [9]. Therefore, PILs are smart materials having the advantages of ILs (enhanced ionic conductivity and high electrochemical stability) combined with excellent processability and good mechanical performances and resulting from the polymeric structure [10,11]. Owing to such unique combination of properties, PILs find applications [12] in diverse fields such as electrochemical supercapacitors [13], catalyst [14], gas adsorption [15], extraction [16,17,18,19], capillary electrochromatography [20], electrochemical sensors [21,22] and fluorescent determination [23]. In recent years, PIL-based materials prepared with IL monomer as modifiers for electrochemical sensors have been found to exhibit high sensitivity for analytes [24,25,26,27]. For example, Wang and co-workers prepared graphene oxide-poly(1-[3-(N-pyrrolyl) propyl]-3-butylimidazolium bromide) and used it as a modifier to construct an electrochemical sensor of bisphenol A detection [24]. In our previous work, we reported an electrochemical sensor with the composite of reduced graphene oxide and poly(1-vinyl-3-ethylimidazolium tetrafluoroborate) for sensitive detection of phenylethanolamine A [26]. Yu et al. utilized the composite of dual hydroxyl-functionalized poly (ionic liquid) and 2,2’-Azinobis-(3-ethylbenzthiazoline-6-sulfonate) as the support to construct a ratiometric electrochemical biosensor for Cu^2+^ determination [25]. 

Although monomer-based PIL composites as modifiers for electrochemical sensors have been extensively investigated [24,25,26,27], cross-linker based PILs or only PILs without any functionalization as modifiers for electrochemical sensors have rarely been studied [28,29,30,31]. This prompted us to carry out the current research, where we prepared a cross-linker-based PIL poly(1,4-butanediyl-3,3′-bis-l-vinylimidazolium dibromide) (poly([V_2_C_4_(mim)_2_]Br_2_)) for sensitive electrochemical detection of 4-NP. Poly([V_2_C_4_(mim)_2_]Br_2_) was synthesized using a simple one-step free-radical polymerization method. The peak current responses of 4-NP using poly([V_2_C_4_(mim)_2_]Br_2_) and a traditional cross-linker ethylene glycol dimethacrylate based poly (EGDMA) modified glassy carbon electrodes were investigated. Apart from selectivity, stability and reproducibility, we applied poly([V_2_C_4_(mim)_2_]Br_2_) sensor for 4-NP detection in environmental samples.

## 2. Materials and Methods 

### 2.1. Materials

4-Nonylphenol and 2,2’-azobisisobutyronitrile (AIBN) were obtained from Aladdin Industrial Corporation (Shanghai, China). Ethylene glycol dimethacrylate (EGDMA), 1-vinylimidazole, 1,4-dibromobutane, 2-nitroaniline, 3-nitroaniline, and 4-nitroaniline were procured from Sigma-Aldrich (St. Louis, MO, USA). IL [V_2_C_4_(mim)_2_]Br_2_ was prepared in our laboratory. Chitosan was procured from Sinopharm Chemical Regent Co., Ltd. (Shanghai, China). Phosphate buffer was prepared using NaH_2_PO_4_ and Na_2_HPO_4_. Deionized water of 18 MΩ cm was applied in the experiments.

### 2.2. Instrumentation

Fourier transform infrared (FT-IR) spectra were obtained with Nexus-470 FT-IR spectrometer (Nicolet, Madison, USA). Surface morphology measurements were performed using a JSM-7500F scanning electron microscope (SEM) (JEOL, Tokyo, Japan). Raman spectra were acquired on a Thermo Scientific DXR2xi Raman spectrometer (Waltham, MA, USA). Thermal gravimetric analysis (TGA) was obtained on a STA-449F3 instrument (Netzsch, Selb, Germany). All electrochemical experiments were accomplished on a CHI660D electrochemical workstation using a three-electrode system, where a modified glassy carbon electrode (GCE) as the working electrode, a platinum wire electrode used as the auxiliary electrode and a saturated calomel electrode (SCE) as the reference electrode.

### 2.3. Preparation of Poly(Ionic Liquid) with [V_2_C_4_(mim)_2_]Br_2_ as Cross-Linker

**Scheme 1:**
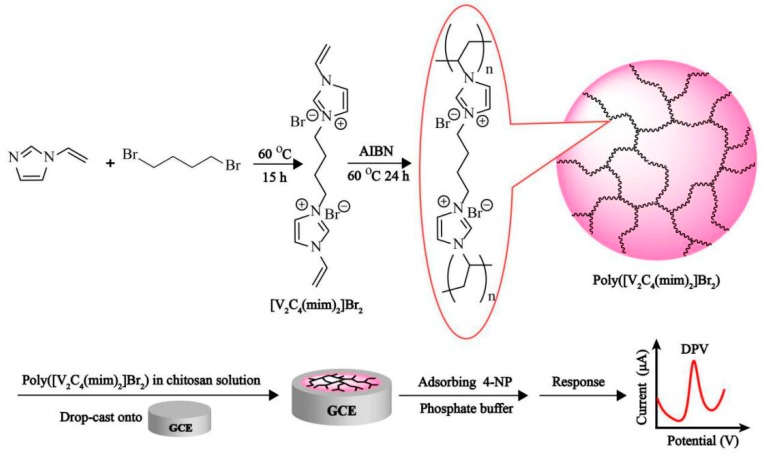
The synthesis route of poly([V_2_C_4_(mim)_2_]Br_2_) and the 4-NP detection process using poly([V_2_C_4_(mim)_2_]Br_2_)/GCE.

Scheme 1 shows the synthesis route of poly([V_2_C_4_(mim)_2_]Br_2_) as a PIL, involving two steps. IL [V_2_C_4_(mim)_2_]Br_2_ was first synthesized from 1-vinylimidazole and 1,4-dibromo butane following a previously published method [30,31]. Poly([V_2_C_4_(mim)_2_]Br_2_) was then prepared using [V_2_C_4_(mim)_2_]Br_2_ as a cross-linker. [V_2_C_4_(mim)_2_]Br_2_ (0.01 mol) and 50 mg of AIBN were added to 30 mL of mixed solvent containing acetonitrile and toluene (30 mL, V_acetonitrile_:V_toluene_ = 1:1). The reaction mixture was purged with nitrogen for 30 min and allowed to polymerize for 24 h at 60 °C. The obtained product was washed with acetonitrile three times and dried at 60 °C under vacuum overnight to obtain a white solid of poly([V_2_C_4_(mim)_2_]Br_2_). For comparison, poly(EGDMA) with traditional cross-linker was synthesized following the same procedure, only replacing [V_2_C_4_(mim)_2_]Br_2_ by EGDMA.

### 2.4. Electrochemical Measurements

Scheme 1 shows the 4-NP detection process using poly([V_2_C_4_(mim)_2_]Br_2_) as an electrochemical modifier. Poly([V_2_C_4_(mim)_2_]Br_2_) (3.0 mg) was dispersed in HAc (1 mL, 1 M) containing chitosan (0.5 wt%) by ultrasonication for 30 min. Each suspension (3.0 μL) was drop-cast onto a cleaned GCE and dried under an infrared lamp. Chitosan was applied as an adhesive to stabilize poly([V_2_C_4_(mim)_2_]Br_2_) onto GCE. The modified GCE were incubated with 4-NP in 0.1 M phosphate buffer (10 mL, pH 6.0) for 6 min and measured by DPV from 0.2 to 0.9 V.

## 3. Results

### 3.1. Characterization of Poly([V_2_C_4_(mim)_2_]Br_2_)

**Figure 1:**
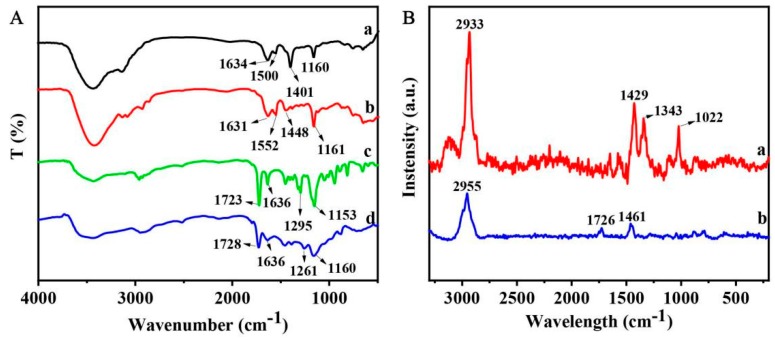
(**A**) FT-IR spectra of [V_2_C_4_(mim)_2_]Br_2_ (a), poly([V_2_C_4_(mim)_2_]Br_2_) (b), EGDMA (c), and poly(EGDMA) (d). (**B**) Raman spectra of poly([V_2_C_4_(mim)_2_]Br_2_) (a) and poly(EGDMA) (b).

**Figure 2:**
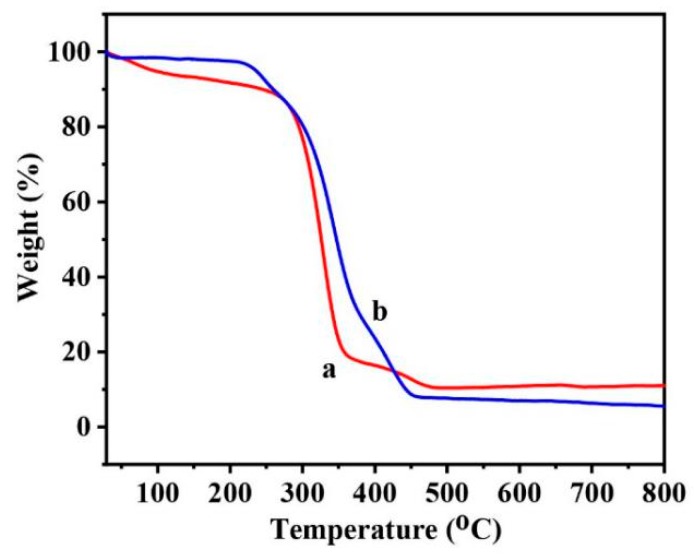
TGA curves of poly([V_2_C_4_(mim)_2_]Br_2_) (a) and poly(EGDMA) (b).

**Figure 3:**
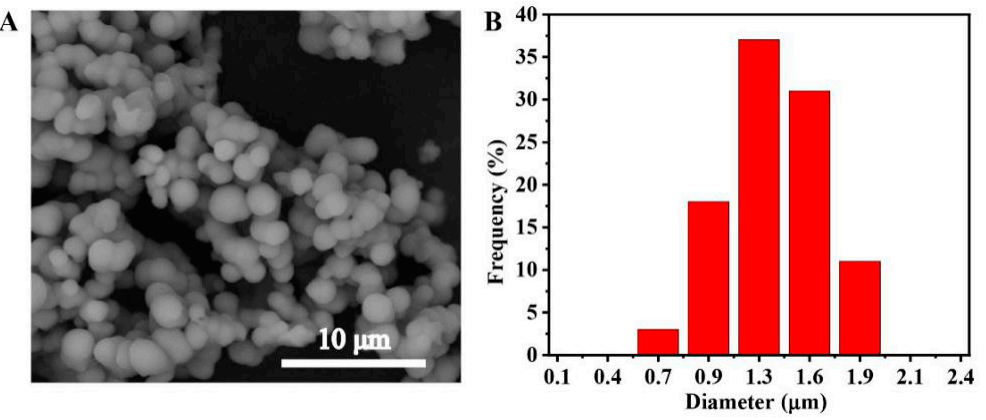
(**A**) SEM image of poly([V_2_C_4_(mim)_2_]Br_2_). (**B**) Size distribution diagram of poly([V_2_C_4_(mim)_2_]Br_2_).

Figure 1A illustrates FT-IR spectra of [V_2_C_4_(mim)_2_]Br_2_, poly([V_2_C_4_(mim)_2_]Br_2_), EGDMA and poly(EGDMA). The peak at 1634 cm^−1^ in the FT-IR spectrum of [V_2_C_4_(mim)_2_]Br_2_ corresponds to exocyclic C=C stretching vibration. The characteristic peaks at 1500, 1401, and 1160 cm^−1^ for [V_2_C_4_(mim)_2_]Br_2_ are attributed to C=N, C=C, and C-N bonds of the imidazolium cation vibrations [32]. The peaks at 1723, 1636, 1295 and 1153 cm^−1^ are ascribed to C-O stretching vibration of carboxylic ester, C=C, C–O stretching vibration of symmetric and asymmetric ester [33], respectively. Poly([V_2_C_4_(mim)_2_]Br_2_) and poly(EGDMA) display similar FT-IR peaks with [V_2_C_4_(mim)_2_]Br_2_ and EGDMA, respectively. Figure 1B shows Raman spectra of poly([V_2_C_4_(mim)_2_]Br_2_) and poly(EGDMA). The peaks at 2933 and 2955 cm^−1^ from Figure 1B were due to CH_2_ stretching vibration [34]. Curve a shows the characteristic vibration bands (1429, 1343, 1022 cm^−1^) corresponding to the imidazolium cation [34], respectively. The peaks at 1726, 1461 cm^−1^ are ascribed to C=O, CH_2_ bonds of poly(EGDMA) (Curve b) [35]. The results show the successful synthesis of poly([V_2_C_4_(mim)_2_]Br_2_) and poly(EGDMA). Figure 2 displays the TGA curves of poly([V_2_C_4_(mim)_2_]Br_2_) and poly(EGDMA). A total weight loss of poly([V_2_C_4_(mim)_2_]Br_2_) and poly(EGDMA) are 88.9% and 94.4% at 800 °C, respectively. The results suggests the thermal instability of poly([V_2_C_4_(mim)_2_]Br_2_) and poly(EGDMA). Figure 3 presents the SEM image of poly([V_2_C_4_(mim)_2_]Br_2_), revealing spherical morphology with diameter ranging from 0.65 to 1.85 μm. 

### 3.2. Electrochemical Behavior of Poly([V_2_C_4_(mim)_2_]Br_2_)

**Figure 4:**
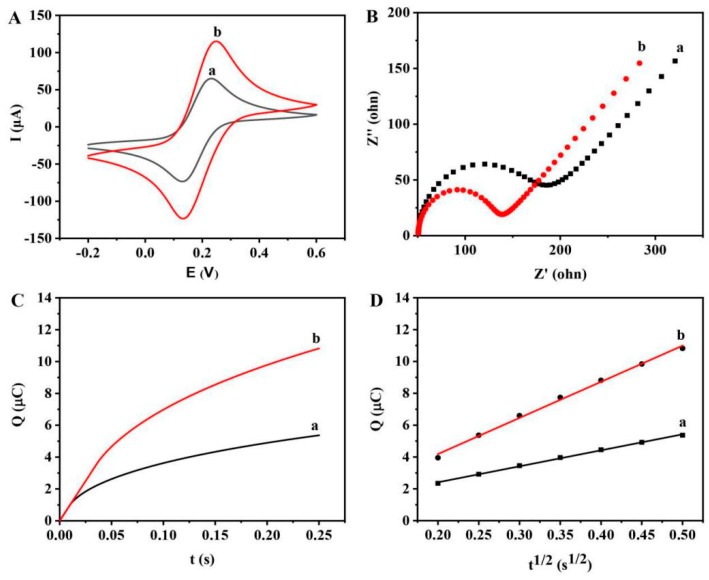
(**A**) Cyclic voltammograms of bare GCE (a) and poly([V_2_C_4_(mim)_2_]Br_2_)/GCE (b) in 0.1 M KCl containing 5.0 mM [Fe(CN)_6_]^3−/4−^. (**B**) Nyquist plots of bare GCE (a) and poly([V_2_C_4_(mim)_2_]Br_2_)/GCE (b) in 0.1 M KCl containing 5.0 mM [Fe(CN)_6_]^3−/4−^. (**C**) Plot of bare GCE (a) and poly([V_2_C_4_(mim)_2_]Br_2_)/GCE (b) in 5.0 mM [Fe(CN)_6_]^3−/4−^. (**D**) Plot of Q-t^1/2^ curves for bare GCE (a) and poly([V_2_C_4_(mim)_2_]Br_2_)/GCE (b) in 5.0 mM [Fe(CN)_6_]^3−/4−^.

We studied the electrochemical behaviors of the bare GCE and poly([V_2_C_4_(mim)_2_]Br_2_)/GCE using cyclic voltammetry (CV). Figure 4A shows the CVs of the bare GCE and poly([V_2_C_4_(mim)_2_]Br_2_)/GCE in 0.1 M KCl containing 5.0 mM [Fe(CN)_6_]^3−/4−^. Compared with the bare GCE (curve a), poly([V_2_C_4_(mim)_2_]Br_2_)/GCE exhibits higher peak current response, suggesting good electrical conductivity of poly([V_2_C_4_(mim)_2_]Br_2_), which improves the electron transfer kinetics of [Fe(CN)_6_]^3−/4−^ at the PIL electrode. In addition, we studied the electrochemical impedance spectroscopy (EIS) of the two electrodes. The charge transfer resistance (Rct) represents the electron transfer kinetics of a redox probe at the electrode surface. We found lower Rct value of poly([V_2_C_4_(mim)_2_]Br_2_)/GCE when compared with the bare GCE and attributed the results to the good electron transfer conductivity of poly([V_2_C_4_(mim)_2_]Br_2_). 

The effective surface areas of the bare GCE and poly([V_2_C_4_(mim)_2_]Br_2_)/GCE were calculated from the plots of Q vs. t^1/2^, obtained by chronocoulometry (Figure 4C) in 0.5 mM K_3_[Fe(CN)_6_] following the Anson method [36,37]. We obtained the effective surface areas of the bare GCE and poly([V_2_C_4_(mim)_2_]Br_2_)/GCE as 0.0669, and 0.151 cm^2^ from the slopes of Q vs. t^1/2^ plot (Figure 4D). The results demonstrate the ability of poly([V_2_C_4_(mim)_2_]Br_2_) to increase the effective surface area. Therefore, we can infer that the effective surface area of poly([V_2_C_4_(mim)_2_]Br_2_)/GCE can enhance the total adsorption ability for 4-NP, thus increasing the detection sensitivity.

**Figure 5:**
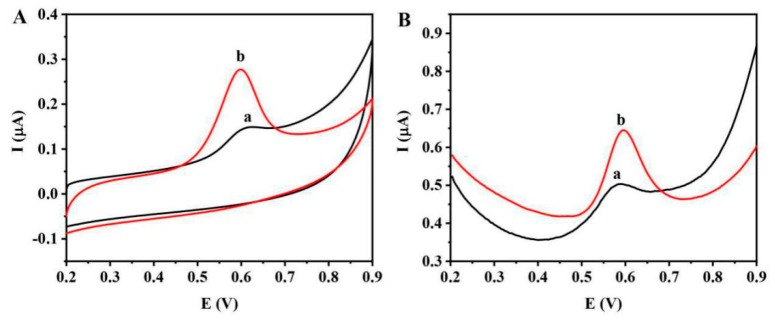
(**A**) Cyclic voltammograms of poly(EGDMA)/GCE (a) and poly([V_2_C_4_(mim)_2_]Br_2_)/GCE (b) after adsorbing 2 μM 4-NP in 0.1 M phosphate buffer (pH = 6.0) for 6 min. Scan rate: 100 mV/s. (**B**) Differential pulse voltammograms of poly(EGDMA)/GCE (a) and poly([V_2_C_4_(mim)_2_]Br_2_)/GCE (b) after adsorbing 2 μM 4-NP for 6 min in 0.1 M phosphate buffer (pH = 6.0).

To highlight the advantage of PIL, we compared the electrochemical responses of poly([V_2_C_4_(mim)_2_]Br_2_)/GCE with poly(EGDMA)/GCE. Poly([V_2_C_4_(mim)_2_]Br_2_)/GCE and poly(EGDMA)/GCE were incubated with a 4-NP solution in phosphate buffer for 6 min, before carrying out the CV and DPV measurements (Figure 5). We noted oxidation of 4-NP on two electrodes (Figure 5A), but no cathodic signal was observed in the scanned potential range. The result indicates an irreversible oxidation process for 4-NP [38]. As shown in Figure 5, poly([V_2_C_4_(mim)_2_]Br_2_)/GCE exhibited higher CV and DPV peak current responses for 4-NP than those of poly(EGDMA)/GCE. The peak current response for 4-NP of poly([V_2_C_4_(mim)_2_]Br_2_)/GCE was 3.6 times than that of poly(EGDMA)/GCE. We ascribed the higher current response to the excellent electrical conductivity and large effective surface area of poly([V_2_C_4_(mim)_2_]Br_2_)/GCE.

### 3.3. Optimization of Experimental Conditions

**Figure 6:**
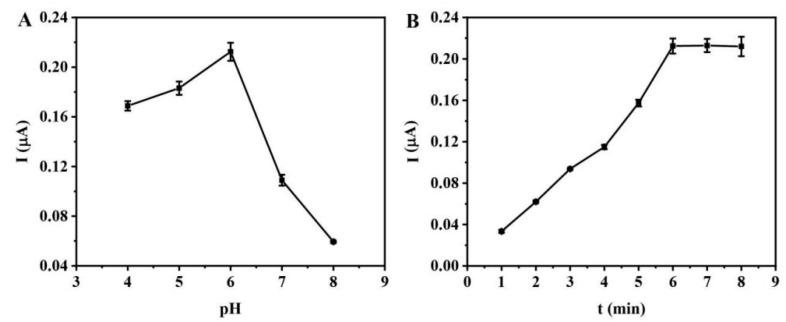
The influence of test solution pH (**A**) and incubation time (**B**) on DPV peak currents at poly([V_2_C_4_(mim)_2_]Br_2_)/GCE after incubating in 2 μM 4-NP in phosphate buffer.

To investigate the influence of pH of the test solution on the current of 4-NP, we measured the DPV of 2 μM 4-NP at the poly([V_2_C_4_(mim)_2_]Br_2_)/GCE over the pH range of 4–8. As shown in Figure 6A, the peak current increased with pH from 4.0 to 6.0; however, we observed a decrease in the peak current beyond pH 6. Hence, we selected pH 6.0 as the optimum pH for subsequent experiments.

Moreover, Figure 6B shows increase in the peak current response from until 6 min before reaching a plateau. This indicates that the poly([V_2_C_4_(mim)_2_]Br_2_)/GCE was saturated by 4-NP adsorption. Thus, further accumulation of 4-NP onto the electrode (beyond 6 min) did not contribute to the enhancement of peak current. Based on our findings, we employed an optimum accumulation time of 6 min for 4-NP adsorption onto the poly([V_2_C_4_(mim)_2_]Br_2_)/GCE in all subsequent experiments.

### 3.4. Analytical Performance of Poly([V_2_C_4_(mim)_2_]Br_2_)/GCE

**Figure 7:**
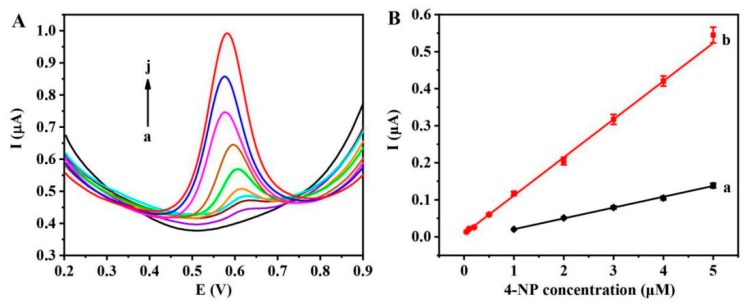
(**A**) Differential pulse voltammograms of poly([V_2_C_4_(mim)_2_]Br_2_)/GCE for 4-NP from 0 (a), 0.05 (b), 0.1 (c), 0.2 (d), 0.5 (e), 1 (f), 2 (g), 3 (h), 4 (i), 5 (j) μM. (**B**) Calibration plots for 4-NP with poly(EGDMA)/GCE (a) and poly([V_2_C_4_(mim)_2_]Br_2_)/GCE (b) (*n* = 3).

Under the optimized experimental conditions, the DPV curves of 4-NP at different concentrations were measured by poly([V_2_C_4_(mim)_2_]Br_2_)/GCE (Figure 7A). We observed a linear increase in the peak current with 4-NP concentration from 0.05 to 5 μM (Figure 7B). We obtained a limit of detection (LOD) of 0.01 μM (S/N = 3) for 4-NP using the following linear regression equation: I(μA) = 0.102 C(μM) + 0.0087 (R^2^ = 0.9980). Compared with the other electrodes reported for 4-NP [1,27,39,40,41,42,43], the poly([V_2_C_4_(mim)_2_]Br_2_)/GCE exhibited satisfactory analytical parameters (Table 1). To demonstrate the advantage of poly([V_2_C_4_(mim)_2_]Br_2_) sensor, we also investigated the linearity of a traditional cross-linker-based poly(EGDMA) sensor for 4-NP (Figure 7B). The poly(EGDMA)/GCE had a detection limit of 0.8 μM (S/N = 3) for a linear 4-NP concentration range of 1.0 to 5 μM. The above findings clearly demonstrate higher sensitivity of poly([V_2_C_4_(mim)_2_]Br_2_) based sensor towards 4-NP when compared with the poly(EGDMA)-based sensor.

### 3.5. Reproducibility, Stability and Selectivity of Poly([V_2_C_4_(mim)_2_]Br_2_)/GCE

To study reproducibility and stability of poly([V_2_C_4_(mim)_2_]Br_2_)/GCE, we performed DPV measurements of 2 μM 4-NP solution using seven modified GCEs prepared independently. We obtained the relative standard deviation (RSD) as 4.35%, confirming good reproducibility of the method. To check the stability, we carried out similar measurements with the poly([V_2_C_4_(mim)_2_]Br_2_)/GCE after storing for two weeks at 4 °C, and found that the sensor could retain 93.7% of its original response (2 μM 4-NP), thus exhibiting good stability.

To evaluate the selectivity, we subjected our poly([V_2_C_4_(mim)_2_]Br_2_)/GCE to detect 4-NP in presence of some possible interfering substances. Table 2 lists the tolerable ratio, which is defined as the maximum amount of foreign species resulting in an error lower than ±5℅ for detecting 2 µM 4-NP. We found that 100-fold of Na^+^, K^+^, Fe^2+^, Mg^2+^, Ni^2+^, Co^2+^, Cu^2+^, Cl^−^, NO_3_^−^, SO_4_^2−^, and 10-fold hydroquinone, catechol, benzene, 1,2-dimethylbenzene, 2-nitroaniline, 3-nitroaniline and 4-nitroaniline had no influence on the determination of 2 µM 4-NP by the poly([V_2_C_4_(mim)_2_]Br_2_)/GCE. 

### 3.6. Analytical Application

Furthermore, we evaluated the validity of the proposed method using the lake water samples collected from Nanhu Lake and rain water samples in Jiaxing city. The environmental water samples were filtered through 0.45 μm filter before use. Since no 4-NP was detected in the water samples by the poly([V_2_C_4_(mim)_2_]Br_2_)/GCE, we applied the standard addition method of spiking the samples with 4-NP for the method validation purpose. As shown in Table 3, the recovery was from 97.3% to 104.0%, and the RSD was less than 4.47%. This suggests the potential of the poly([V_2_C_4_(mim)_2_]Br_2_)/GCE for the detecting 4-NP in environmental water samples. 

## 4. Conclusions

In an attempt to construct a sensitive electrochemical sensor for 4-NP, we synthesized a cross-linker-based PIL of poly([V_2_C_4_(mim)_2_]Br_2_) as the sensing agent via a simple one-step free-radical polymerization method. Poly([V_2_C_4_(mim)_2_]Br_2_)/GCE with a large effective surface area exhibited good electrical conductivity and higher peak current response for 4-NP than that of poly(EGDMA)/GCE, indicating the advantage of poly([V_2_C_4_(mim)_2_]Br_2_). In addition to excellent sensitivity, the poly([V_2_C_4_(mim)_2_]Br_2_) based sensor also demonstrated 4-NP detection in environmental water samples. 

## Figures and Tables

**Table 1:** Comparison with other electrochemical methods for the determination of 4-NP.

Detection Method	Linear Range (μM)	Detection Limit (μM)	Ref.
CTAB ^a^/carbon paste electrode	0.1–25	0.01	[1]
AuNPs/PILs ^b^/GCE	0.1–120	0.033	[27]
β-CD-SH-GR ^c^/Au electrode	0.07–70	0.061	[39]
GR ^d^-DNA/GCE	0.05–4	0.01	[40]
MIL-101(Cr)@Rgo ^e^/GCE	0.1–12.5	0.033	[41]
MIP ^f^/AuNPs/TiO_2_/GCE	0.95–480	0.32	[42]
IL-FGNS ^g^/GCE	0.5–200	0.058	[43]
Poly([V_2_C_4_(mim)_2_]Br_2_)/GCE	0.05–5	0.01	This work

^a^: Cetyltrimethylammonium bromide; ^b^: Poly(1-butyl imidazole-3-(2-ethyl methacrylate) tetrafluoroborate) ionic liquid hollow nanospheres; ^c^: Thiol-β-cyclodextrin (β-CD-SH) and graphene (GR) hybrid; ^d^: Reduced graphene; ^e^: Chromium terephthalate metal-organic frameworks@reduced graphene oxide; ^f^: Molecularly imprinted polymer; ^g^: Ionic liquid-functionalized grapheme nanosheet.

**Table 2:** Effects of foreign species on the determination of 4-NP.

Foreign Species	Tolerable Ratio
Na^+^, K^+^, Fe^2+^, Mg^2+^, Ni^2+^, Co^2+^, Cu^2+^, Cl^−^, NO_3_^−^, SO_4_^2−^	100
Hydroquinone, catechol, benzene, 1,2-dimethylbenzene, 2-nitroaniline, 3-nitroanilie, 4-nitroaniline	10

**Table 3:** Detection of 4-NP in water samples by poly([V_2_C_4_(mim)_2_]Br_2_)/GCE (*n* = 3).

Sample	Added 4-NP (μM)	Found 4-NP (μM)	Recovery (%)	RSD (%)
Lake water	0.1	0.104	104.0	4.38
	1	0.991	99.1	4.47
	2	1.946	97.3	3.45
Rain water	0.1	0.0982	98.2	3.24
	1	1.031	103.1	4.43
	2	2.026	101.3	4.04
